# Hemoptysis following Talc Pleurodesis in a Pneumothorax Patient

**DOI:** 10.1155/2017/5846242

**Published:** 2017-10-16

**Authors:** Yusuke Kakiuchi, Fumihiro Yamaguchi, Makoto Hayashi, Yusuke Shikama

**Affiliations:** Department of Respiratory Medicine, Showa University Fujigaoka Hospital, 1-30 Fujigaoka, Aoba-ku, Yokohama 227-8501, Japan

## Abstract

The purpose of this article is to report a case of hemoptysis occurring in combination with secondary spontaneous pneumothorax following chemical pleurodesis by talc. A Japanese male with cancer of renal pelvis was found with the left pneumothorax and multiple lung metastases. A computed-tomography scan revealed severe emphysema throughout the lungs. Talc pleurodesis was employed to arrest air leakage. The patient developed hemoptysis 45 minutes after talc injection into the thorax. This is the first report of hemoptysis following talc pleurodesis. The agent could induce severe inflammation in capillary vessels of the lung following visceral pleura infiltration.

## 1. Introduction

Spontaneous pneumothorax is a common condition divided into primary and secondary types on the basis of presence or absence of underlying lung diseases such as emphysema and interstitial pneumonia [1, 2]. More specifically, secondary spontaneous pneumothorax occurs especially in elderly patients and causes severe respiratory failure in some cases. Surgical intervention is the procedure of choice in patients with intractable pneumothorax. However, chemical pleurodesis is useful for more elderly patients who generally exhibit poor lung function. The efficacy of chemical pleurodesis in pneumothorax patients is well established in clinical practice [3, 4], and talc is the substance most commonly used worldwide despite the occurrence of severe side effects including acute respiratory distress syndrome (ARDS) [5–7]. This report details the occurrence of hemoptysis in combination with secondary spontaneous pneumothorax following talc pleurodesis, suggesting that talc induced severe inflammation in capillary vessels of the lung.

## 2. Case Report

**Figure 1:**
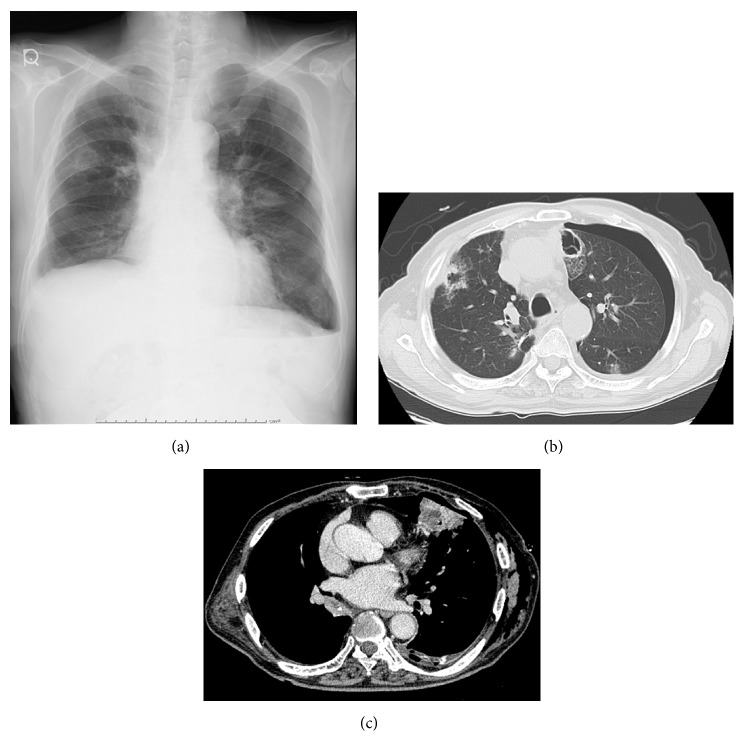
(a) A chest X-ray showed a left pneumothorax and multiple metastases in the bilateral lung. (b) A CT scan of the chest revealed that some nodules with cavities were located in the edge of the lung. (c) A contrast enhanced CT scan of the chest revealed a cavity, culprit lesion of hemoptysis, 25 mm in size, in the left upper lobe.

**Figure 2:**
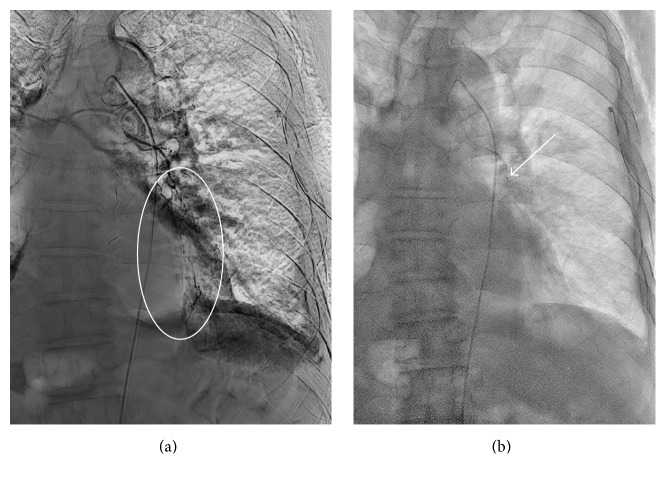
(a) A chest X-ray showed bleeding from the left bronchial artery in the left upper lobe (white circle). (b) A chest X-ray indicated the arrest of bleeding after bronchial artery embolization (white arrow).

A 72-year-old Japanese male with cancer of the renal pelvis and a smoking history of 120 pack-years had continued on two cycles of doublet chemotherapy consisting of cisplatin with gemcitabine for the past year. He was referred to our hospital because of acute dyspnea. A computed-tomography (CT) scan of his chest at diagnosis revealed left pneumothorax, multiple nodules, pleural effusion, and severe emphysema throughout the lungs.  Figure 1 shows that some nodules with cavities were located in the edge of the lung. As shown in Table 1, tumor markers CA19-9, SCC, and CYFRA21-1 were elevated with values of 903.4 U/ml, 9.5 ng/ml, and 23.6 ng/ml, respectively. Peripheral blood count was consistent with mild anemia and coagulation parameters were within normal limits. He was therefore diagnosed with lung metastasis. A chest tube was inserted into the seventh intercostal space in the left median axillary line for drainage on day 1; however air leakage continued. Subsequently 70 ml of autologous blood was injected into the left thorax through the chest tube on day 12, but this intervention was unsuccessful. Poor lung function was inferred due to the severe emphysema and surgical intervention was thus deemed inappropriate. In addition left pleural effusion was detected, and it was possible that such effusion could have been increased by lung metastasis. Therefore talc pleurodesis was employed to arrest air leakage and prevent recurrent pleural effusion. 50 ml of saline containing 4 g sterile talc powder was administered into the left thorax on day 17. The patient developed hemoptysis 45 minutes after talc injection through the chest tube and immediately required a bronchial artery embolization (Figure 2). Talc pleurodesis was eventually successful and the chest tube was then removed.

## 3. Discussion

Surgical intervention is generally applied for recurrent or complicated pneumothorax. However, chemical pleurodesis is useful in management of inoperable patients. In fact intrapleural adhesion has been described as a treatment for patients with secondary spontaneous pneumothorax according to the clinical guidelines published by the British Thoracic Society [3] and the American College of Chest Physicians [4]. Agents that reportedly induce intrapleural adhesions include tetracycline derivatives, bleomycin, cisplatin, iodopovidone, 50% dextrose in water, autologous blood, and OK-432 [8–12]. In particular, talc is the most commonly used such agent worldwide because of its high efficacy and low cost [13]. Complications of talc pleurodesis were initially reported due to the presence of contaminants such as asbestos [14], but talc in current use is purified and sterilized and its efficacy has been well established [15]. There have been several reports that talc administration into the thorax is more effective in preventing recurrent pneumothorax or reducing malignant pleural effusion than other sclerosing agents [6, 7]. In the present case, both left pneumothorax and pleural effusion were detected. Hence talc was selected to arrest air leakage and prevent recurrent pleural effusion following failed pleurodesis with autologous blood. On the other hand it should be noted that intrapleural talc administration can lead to severe side effects. More specifically, many physicians have reported ARDS associated with talc pleurodesis [16–18]. In the present case the patient did not exhibit symptoms of ARDS but developed hemoptysis shortly after establishment of intrapleural adhesion. This is the first report of hemoptysis following talc pleurodesis, whereas hemothorax has previously been reported in a few cases [1]. Such side effects can be related to the size and dose of talc in the preparation. Several previous studies have shown that intrapleural adhesion with low doses of talc (<2-3 g and medium particle size > 6–31.5 *μ*m) did not induce either ARDS or pneumonitis [19, 20]. In a rabbit model, IL-8 and VEGF levels in serum were elevated with decreasing talc particle size; furthermore pleural fluid IL-8 and VEGF levels were higher in the small particle talc group (<5 *μ*m) than in particles of mixed size [21]. Moreover talc deposition in the lung was observed more clearly with small size particles. Talc particles were attached not only to the pleural surface, but also to the alveolar spaces and septa in the lung which was the site of injection with talc. Conversely, talc deposition was detected only in alveolar septa in the opposite lung. In addition, particle migration was demonstrated in spleen, liver, and kidney which were vascularized [21]. These findings suggest that talc circulates throughout the body and can be deposited on capillary vessel walls. As a result, these particles could damage endothelial cells directly, or by producing inflammatory mediators, and thereby develop hemoptysis. In the present case, the patient vomited blood only 45 minutes after talc injection. It is not clear how much time is required for talc particles to infiltrate the pleural surface through mesothelial cells. As shown in Figure 1, cavity-forming cancer nodules were adjacent to visceral pleura, suggesting the fragile nature of the pleural barrier. An agent could therefore penetrate mesothelial cells on the pleural surface relatively quickly and cause endothelial injury in the cavity. Indeed it has been reported that talc exposure induces prominent damage to mesothelial cells within 15 minutes [22]. In the current study, a bronchial artery embolization was performed for the arrest of bleeding, and it revealed that the culprit vessel was a bronchial artery (Figure 2). There was no formation of a communication between bronchial arteries and pulmonary arteries.

There is no relationship between the success rate of pleurodesis and talc dosage over the range >2–10 g [23]. Low dose talc should therefore be considered, particularly for elderly patients, because dose reduction can potentially suppress adverse effects. In addition small particles of talc could be associated with an increased risk of severe side effects such as ARDS and hemoptysis, and commercial talc is a heterogeneous material whose mean particle size may vary, depending on the location from which it is sourced [24]. The results of this study stress the need for careful attention in patients with secondary spontaneous pneumothorax when using talc for production of intrapleural adhesions.

## Figures and Tables

**Table 1:** Laboratory data.

Peripheral blood counts	
WBC	9600/*µ*l
RBC	418 × 10^4^/*µ*l
Hb	12 g/dl
Plt	23.4 × 10^4^/*µ*l

Blood coagulation	
PT	126%
APTT	>120%

Tumor marker	
CA19-9	903.4 U/ml
SCC	9.5 ng/ml
CYFRA21-1	23.6 ng/ml
